# Recent Progress in MEMS Fiber-Optic Fabry–Perot Pressure Sensors

**DOI:** 10.3390/s24041079

**Published:** 2024-02-07

**Authors:** Ye Chen, Dongqin Lu, Huan Xing, Haotian Ding, Junxian Luo, Hanwen Liu, Xiangxu Kong, Fei Xu

**Affiliations:** 1 MIIT Key Laboratory of Aerospace Information Materials and Physics, State Key Laboratory of Mechanics and Control for Aerospace Structures, College of Physics, Nanjing University of Aeronautics and Astronautics, Nanjing 211106, China; yechen@nuaa.edu.cn; 2 National Laboratory of Solid State Microstructures, College of Engineering and Applied Sciences, Nanjing University, Nanjing 210093, China

**Keywords:** fiber-optic devices, pressure sensor, Fabry–Perot, MEMS

## Abstract

Pressure sensing plays an important role in many industrial fields; conventional electronic pressure sensors struggle to survive in the harsh environment. Recently microelectromechanical systems (MEMS) fiber-optic Fabry–Perot (FP) pressure sensors have attracted great interest. Here we review the basic principles of MEMS fiber-optic FP pressure sensors and then discuss the sensors based on different materials and their industrial applications. We also introduce recent progress, such as two-photon polymerization-based 3D printing technology, and the state-of-the-art in this field, e.g., sapphire-based sensors that work up to 1200 °C. Finally, we discuss the limitations and opportunities for future development.

## 1. Introduction

Pressure sensing plays an important role in many industrial fields, such as human–machine interactive devices [1,2], electronic skin [3], endoscopic instruments [4], and aerodynamic measurements [5,6], etc. Conventional electronic pressure sensors based on piezoresistive [6,7,8], piezoelectric [9,10], and capacitive [4,11,12] principles struggle to survive in harsh environments, as they still suffer electromagnetic interference, large temperature cross-sensitivity, and poor corrosion resistance. 

Fiber-optic-based sensors [13,14,15,16,17,18,19,20,21,22,23], which can overcome the shortcomings of electrical sensors due to the advantages of their anti-electromagnetic interference, flexibility, and corrosion resistance, have received extensive attention from researchers. Common fiber-optic pressure sensors include point type, such as long period grating [15,16], fiber Bragg grating (FBG) [17,18,19,20], fiber Mach–Zehnder (MZ) interferometer [21,22,23], fiber FP interferometer [24], and so on [25,26]. Among them, the MEMS fiber-optic FP pressure sensor is the focus of researchers thanks to its simple structure, small size, high accuracy, low temperature crosstalk, and resistance to complex environments.

For different application scenarios, a large number of new MEMS fiber-optic FP pressure sensors based on a variety of materials and structures have been proposed, such as sapphire FP sensors for aerospace scenarios [27], ultra-small-size flexible pressure sensing for medical endoscopy applications [28], and so on. Meanwhile, with the expansion of applications, many scientific and technical issues also require further in-depth study by researchers, including both individual issues, e.g., new materials for high-temperature scenarios, as well as common issues, e.g., methods for reducing temperature disturbances.

Here we review the basic principles of MEMS fiber-optic FP pressure sensors with different materials and industrial applications. We also introduce the recent progress and state-of-the-art in this field and discuss the limitations and opportunities for future development.

## 2. The Principles of MEMS Fiber-Optic FP Pressure Sensors

**Figure 1:**
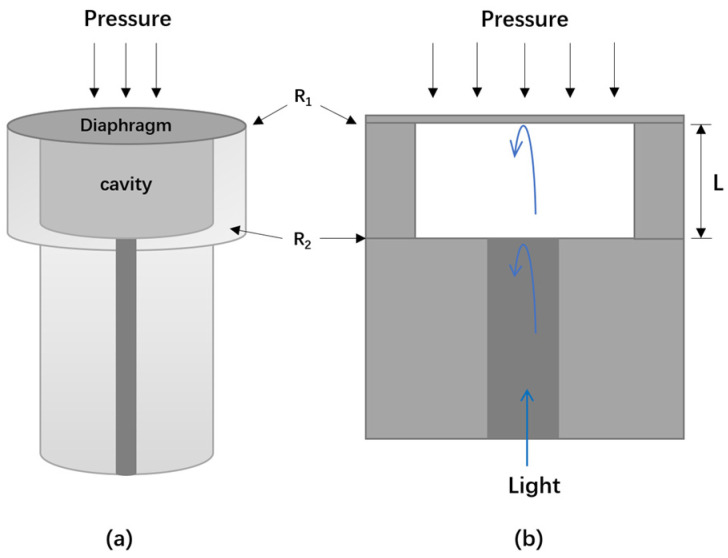
(**a**) Schematic of MEMS fiber-optic FP pressure sensors; (**b**) cross-section of the cavity structure.

Figure 1 illustrates the structure of a typical MEMS fiber-optic FP pressure sensor. The main working principle of this type of sensor is FP interference, which converts external pressure changes into FP cavity length changes through a sensitive diaphragm. The inner surface of the diaphragm (R_1_) and the end face of the fiber (R_2_) constitute two cavity surfaces of the FP cavity. The two-beam interference equation can be shown as:

(1) $$I_{R}(\lambda )=I_{1}+I_{2}+2\sqrt{I_{1}I_{2}}cos(\Delta \varphi +\varphi_{0})$$

(2) $$\Delta \varphi =\frac{4\pi nL}{\lambda }$$

where $I_{1}$ and $I_{2}$ are the intensities reflected by R_1_ and R_2_, respectively; n is the refractive index of the cavity material; L is the length of the cavity; $\varphi_{0}$ represents the initial phase; and Δφ is the optical phase difference between the two reflected light beams.

According to the elastic deformation principle of thin film, the pressure-sensitive diaphragm deforms under external pressure, thus changing the length of the FP cavity and causing the change of the interference spectrum of light from reflecting surface 1 and reflecting surface 2 (R_1_ and R_2_). The relationship between pressure change and cavity length can be expressed by the following formula [29]:

(3) $$\Delta L=\frac{3(1-\nu^{2})R^{4}}{16Ed^{3}}\Delta p$$

where ν and E are Poisson’s ratio and Young’s modulus of the diaphragm material, respectively; R is the radius of the diaphragm; d is the diaphragm thickness; and Δp is the ambient pressure change.

From the above principles we can see that the design and fabrication of the FP cavity of the fiber-optic pressure sensor is a key factor in determining the pressure sensitivity. Among them, the shape, diameter, thickness, and hardness of the pressure-sensitive membrane determine the optical path sensitivity; thus, to improve the pressure sensitivity, there are several methods including: (1) increasing the sensing area; (2) using a more flexible material; (3) thinning the thickness of the diaphragm. Increasing the sensing area is a relatively easy way, but a larger area will reduce the mechanical resonance frequency of the film, which, in turn, weakens the high-speed response characteristics of the device. Replacing flexible materials such as polymers can result in higher sensitivity for the same sensing area. However, polymer materials are often difficult to meet the application requirements in terms of high temperature resistance and other characteristics. Therefore, in recent years, the thinning process of thin films has become a hotspot of researchers’ attention.

In addition, one of the main factors affecting the performance of the device is the interference of temperature. The temperature cross-sensitivity mainly comes from two reasons: the residual gas expansion induced force applied on the inside face of the diaphragm and the thermal stress caused by thermal expansion mismatch between the diaphragm and cavity body materials. To overcome the temperature–pressure cross-error, compensation can be achieved through device design, processing improvements, or by utilizing real-time temperature monitoring.

## 3. Different Materials of MEMS FP Pressure Sensor Diaphragms

The sensor pressure-sensitive diaphragms are made of different materials, of which there are two categories:

1. Dielectric films such as silica, silicon, sapphire, silicon carbide (SiC), etc.;
2. Non-dielectric membranes, including metallic membranes, e.g., silver, gold; polymers, e.g., polydimethylsiloxane (PDMS), polyvinyl chloride (PVC), etc.; and two-dimensional material (2D) materials, e.g., graphene, molybdenum disulfide (MoS_2_), etc.

The following is a detailed review of recent developments based on the selection of different diaphragm materials.

### 3.1. Dielectric Diaphragm

#### 3.1.1. Silica Diaphragm

The silica diaphragm-based MEMS fiber-optic FP pressure sensor has the advantage of compatibility with the fiber-optic process technique. The raw materials for its processing are mainly a variety of commercial silica fibers, and the processing equipment is a fusion splicer and other common optical fiber processing equipment, thus bringing the advantages of simplicity to process, low-cost, and small size. Ultra-small size all-silica sensors can be easily realized to meet the requirements of specific scenarios such as aerospace high-temperature applications, medical endoscopy, and so on.

The preparation of silica film-based MEMS fiber-optic FP pressure sensors includes fusion splicing, cleaving, wet chemical etching, and other steps [30,31,32]. The air cavity can be obtained by wet etching a large-core multimode optical fiber or directly by fusing and cutting a hollow optical fiber. Cavity length determines the spectral properties of the FP. Researchers often cut with the assistance of a microscope for precise control of the cavity length. The processing of thin film is mainly realized by the cutting, polishing, and etching of optical fiber. Normally, polishing can achieve a thickness of the diaphragm of ~3–5 μm. To further reduce the thickness, the wet etching technique needs to be used. As the diameter and thickness of the film determine the sensitivity of the sensor, researchers have made many efforts to precisely control the polishing and etching process. Donlagic et al. [30] presented online monitoring during the diaphragm etching, achieving 3.4 nm/kPa sensitivity.

**Figure 2:**
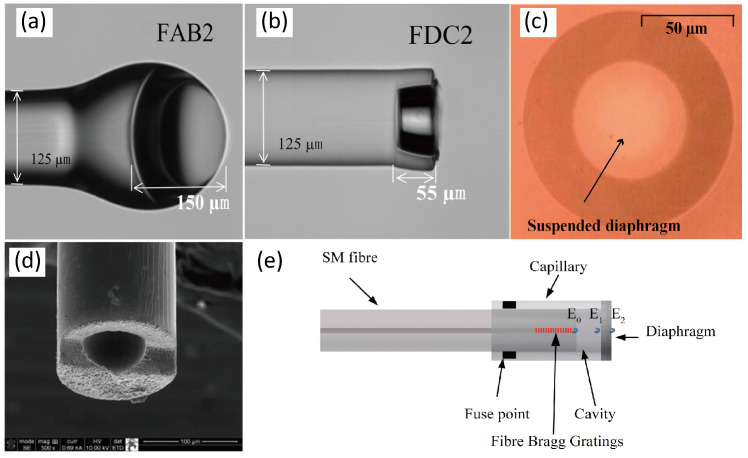
Optical microscopy image of (**a**) fiber-tip air bubble(FAB) specimen FAB2 with a cavity length of about 155 μm and a diaphragm thickness of about 290 nm; (**b**) fiber-tip diaphragm-sealed cavity(FDC) specimen FDC2 with a cavity length of about 55 μm and a diaphragm thickness of about 250 nm (reprinted with permission from Ref. [34] © Springer Nature, 2017); (**c**) diaphragm fabricated by etching away the silicon substrate (reprinted with permission from Ref. [37] © Optical Society of America); (**d**) femtosecond laser fabricated fiber-optic FP structure (reprinted with permission from Ref. [39] © Optical Society of America); (**e**) FBG integrated structure for temperature compensation. The pressure sensor was fabricated by splicing the polished capillary and a multimode fiber (MMF), which forms a diaphragm. The single mode fiber (SMF) with FBG was cleaved and inserted into the capillary (reprinted with permission from Ref. [40] © MDPI, 2017).

In order to further reduce the thickness of the film and improve the sensitivity of the device, microbubble-based processes have been induced in recent years, as illustrated in Figure 2a,b [33,34,35,36]. The diaphragm is fabricated using an electrical arc discharge technique and then transferred to the front of the FP cavity by means of fusion bonding. The technology enables films hundreds of nanometers thick, which greatly enhances sensor sensitivity. Liu et al. [34] presented an ultrathin silica diaphragm with a thickness of 170 nm and a pressure sensitivity of 24.44 nm/kPa.

Although the above methods can be easily realized in the laboratory by fiber equipment such as splicers, offering low cost and high sensitivity, they rely heavily on manual labor and are not suitable for batch processing. In order to solve this problem, researchers introduced the MEMS process to realize the ultrathin silica films to improve the consistency and sensitivity of the devices [37,38]. The cavity of the sensor is still fabricated by using the optical fiber method, but the silica film is prepared separately and finally bonded to the cavity. In 2010, Wang et al. [37] realized a 3-μm thick silica diaphragm fabricated by etching away the silicon substrate, as shown in Figure 2c. The fiber-optic sensor showed 1.16 nm/kPa. In 2019, Guo et al. [38] presented a sensor based on a 1.2 μm thick silica diaphragm and accomplished 12.4 nm/kPa.

In recent years, researchers have also developed a series of laser micromachining techniques based on femtosecond or other lasers [39,41]. In 2009, Ran et al. [41] proposed an all-fiber in-line tip pressure sensor fabricated by 157 nm laser pulses. Due to the cold processing characteristic of the femtosecond laser, high-precision cutting, thinning, and roughening processes can be realized. Therefore, it is expected to realize a one-stop processing solution, avoiding excessive processing procedures and environmental pollution by means of wet etching. Meanwhile, a femtosecond laser has been successfully applied to grating writing, which can be used for temperature compensation. In 2013, Zhang et al. [39] demonstrated a fiber-optic FP pressure sensor fabricated by a femtosecond laser (Figure 2d).

Pressure measurement accuracy is affected by many aspects, of which temperature crosstalk is the main factor. The common method is to use FBG as an in situ temperature sensor to realize temperature–pressure cross-decoupling. In 2017, Duraibabu et al. [40] presented MMF-based FP combined with an FBG, as shown in Figure 2e. In addition, the thickness of the film has a great relationship with the linearity of the pressure response; the thicker the device, the higher the linearity of the sensor pressure response. From the experiment results of Huang et al. [42], the pressure response of the 4.63 μm thick diaphragm shows good linearity while the 2.09 μm thick sample shows poor linearity.

For pure silica fiber-optic FP pressure sensors, there is no internal stress caused by material thermal expansion coefficient mismatch, and thanks to precise control of cavity length and film thickness, as well as high-temperature fusion bonding, the devices have the advantages of strong firmness, high sensitivity, and resistance to high temperatures and high pressures. However, high-temperature bonding limits the application of processes such as coating during processing, and the batch processing of devices is also a major challenge.

#### 3.1.2. Silicon Diaphragm

The biggest advantage of the lithography process is the batch processing capability. Silicon is the most commonly used material for MEMS, and it is easy to realize the precision control of film thickness and area through the MEMS lithography process. At the same time, the high reflectivity of silicon can effectively improve the optical characteristics of the device without coating. Therefore, the MEMS fiber-optic FP pressure sensors based on silicon thin films have been a concern of researchers.

The processing flow of the device includes lithography-based cavity processing and cavity and fiber integration. In order to realize the FP sensor structure, the most critical processes are the processing and bonding of the film and the cavity body. The processing of the silicon film is often realized based on chemical mechanical polishing (CMP) and etching, while the choice of materials for the cavity includes glass, silicon, etc. Packaging integration of the cavity with the optical fiber is generally performed by a gluing or bonding technique.

Conventional silicon diaphragm-based FP cavities are made of Pyrex glass and assembled using an anodic bonding process, which has the advantage of low-temperature bonding processing [43,44,45,46,47]. The pressure sensitivity can be easily adjusted by designing the thickness of the silicon diaphragm and the microcavity diameter. In 2006, Li et al. [43] demonstrated a glass–silicon structured MEMS FP for pressure sensing. The sensitivity is experimentally characterized to be 10.07 nm/MPa (spectrum shift/pressure). However, the temperature dependency of the sensor is not addressed.

Meanwhile, in order to eliminate temperature interference, researchers have developed various techniques. Vacuum bonding can be introduced in the process to eliminate the effect of air expansion and reduce the temperature sensitivity of the FP cavity [44,45]. In addition, since some gases are released during anodic bonding, which will affect the effect of vacuum bonding, researchers have developed metal bonding such as Au/Au thermal compression bonding [46]. In addition, researchers have also developed techniques including dual FP [44], fiber grating [45], or temperature-sensitive fluorescent materials [47] for in situ temperature measurement. In 2014, Yin et al. [44] presented a silicon–glass–silicon sandwich structure, constructing two serially connected FP cavities. The first cavity is a silicon cavity to achieve temperature sensing, while the second cavity is a vacuum cavity and employs a thin silicon diaphragm as a pressure-sensing element. In 2018, Jia et al. [45] realized an FBG as the temperature sensor, and Jiang et al. [47] presented temperature-sensitive fluorescent materials for temperature decoupling in 2019.

**Figure 3:**
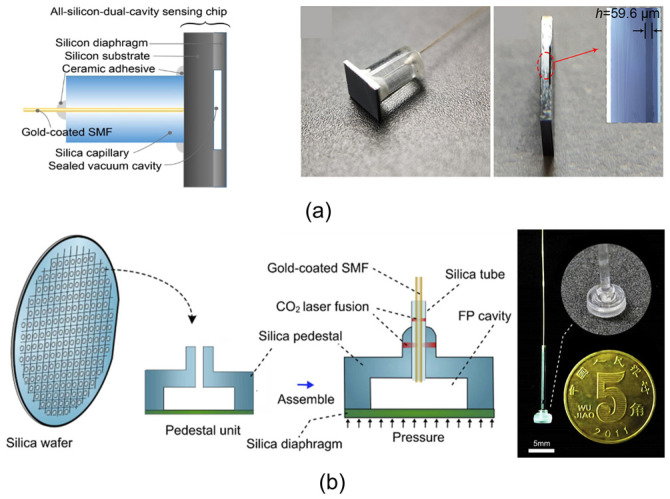
(**a**) (**Left**) schematic diagram of the all-silicon-based dual-cavity fiber-optic pressure sensor structure; (**middle**) complete sensor after the MEMS process and package; (**right**) sectional view of a sensing chip and the inset is the detailed section view under a microscope (reprinted with permission from Ref. [48] © Chinese Laser Press, 2021); (**b**) (**left**) schematic of the proposed batch-produced all-silica pressure sensor. The all-silica pressure sensor comprises a sensor head formed by a silica diaphragm and a silica pedestal, hollow silica tube, and gold-coated SMF; (**right**) all-silica pressure sensor assembled using CO_2_ laser; inset details the fusion using CO_2_ laser. Scale bar: 5 mm (reprinted with permission from Ref. [49] © Elsevier, 2022).

Though a 125 μm diameter sensor based on a Pyrex glass cavity can be achieved; the mismatch of the thermal expansion coefficients of Pyrex glass and a silicon diaphragm tends to introduce internal stresses, thus affecting the performance of the device. In addition, due to the low softening temperature of Pyrex glass, the anodic-bonded Pyrex glass–silicon structure cannot work properly when the temperature is above 350 °C; thus the devices are difficult to apply in extreme environments with high temperatures. Therefore, the researchers have developed an all-silicon FP cavity processing process, where the cavity and diaphragm are prepared by a mask etching and high-temperature wafer bonding process. Wang et al. [48] demonstrated an all-silicon dual-cavity fiber-optic pressure sensor based on direct bonding in 2021, as illustrated in Figure 3a. The presented sensor can survive under high temperatures up to 700 °C. In addition, the silicon layer of the substrate can be used as a solid FP for temperature compensation. Some researchers have also utilized silica wafers instead of glass and silicon, which are still processed based on the MEMS lithography process to improve the device’s working temperature. Li et al. [49] presented a sensor working up to 800 °C in 2022, which was fabricated by silica wafer high-temperature direct bonding technology, as shown in Figure 3b.

In addition, in order to adapt to the application of high temperatures and other extreme environments, researchers have further developed the cavity and fiber integration process. Traditional integration methods often use UV glue or epoxy resin materials to bond the optical fiber, ferrule, and cavity body together, and the temperatures the glues can withstand are generally less than 500 °C. By the application of a high-temperature ceramic adhesive [48] or a CO_2_ laser welding process [45,49], the connection part of the optic fiber and cavity body can withstand temperatures higher than 500 °C.

Up to now, most of the reported all-silicon MEMS FP cavities show large sizes and thick diaphragms, which are mainly limited by the processing and cutting technique. The typical device diameter and device height are 1–10 mm [48,49], compared with the 125 μm diameter of the fiber. The large volume limits the application of the device to scenarios requiring small sensor sizes. Also, the mass of the cavity will cause the bonding point to be easily damaged in a strong vibration environment. In order to achieve a smaller size, further development of the processing and dicing techniques is needed.

#### 3.1.3. Sapphire Diaphragm

**Figure 4:**
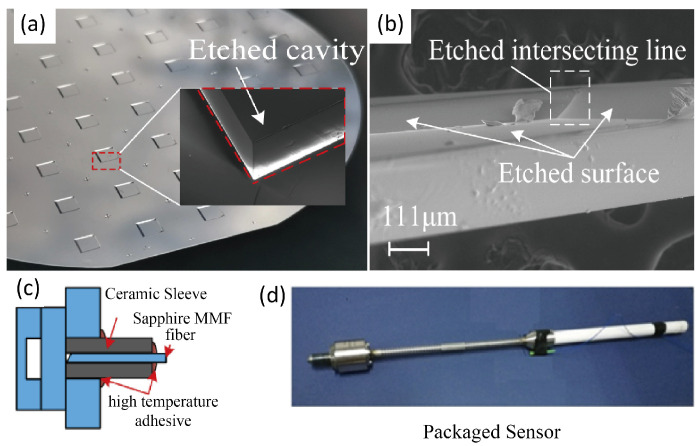
(**a**) Photos of wet etched sapphire wafer; (**b**) the cross sectional SEM image of an etched sapphire cavity (reprinted with permission from Ref. [53] © Optical Society of America); (**c**,**d**) schematic and photo of sapphire diaphragm-based fiber-optic pressure sensors and the packaged device. The FP sensor in (**c**) was fabricated by etching sapphire diaphragm, direct bonding sealed-cavity and glued with sapphire MMF. The FP sensor was finally encapsulated inside a specially customized metal tube to package the sensor to resist the high temperature and pressure (**d**) (reprinted with permission from Ref. [54] © Optical Society of America).

Due to the creep temperature of silicon, Pyrex glass, and silica are lower than 1000 °C, the above FP pressure sensors are difficult to apply in harsh environments such as turbine engines, high-speed aircraft, and other aerospace applications. Sapphire has a high melting temperature up to 2050 °C and a wide transmission spectral range. The development of sapphire-based fiber-optic FP pressure sensors has been a long-term concern of researchers [50,51,52,53,54,55]. Figure 4 illustrates the recent progress of sapphire diaphragm-based fiber-optic FP sensors.

The common fabrication method is to assemble separate sapphire elements of the cavity. Diaphragms are generally obtained by polishing single-crystal sapphire slices, which are then integrated with the cavity body through high-temperature bonding. The encapsulated housing is generally manufactured with high-temperature-resistant materials such as stainless steel or zirconia ceramic inserts. Finally, a high-temperature ceramic adhesive is utilized to bond the optical fiber to the cavity. In order to achieve temperature compensation, a dual-FP structure is usually utilized to achieve cross-decoupling through a temperature-sensitive solid FP. In 2020, Wang et al. [50] realized an adhesive-free encapsulation sapphire FPI sensor which can work up to 1200 °C.

In order to reduce the device size and improve the device’s structural firmness and strength, researchers have been striving to develop an all-sapphire wafer-based FP cavity by the MEMS lithography process and direct bonding technique [51,52,53,54]. The fabrication method provides precise control of the film thickness by means of the silicon oxide-masked wet or inductively coupled plasma (ICP) etching process. The integration of the FP film and the cavity is realized by means of high-temperature direct bonding. In addition, a dual-FP structure can be easily introduced for temperature compensation. In 2021, Shao et al. [53] proposed a cascaded all-sapphire dual-FP sensor based on wet etching and the direct bonding process. The device can work in an 25–800 °C environment. Then, in 2022, Sapphire MMF was used to replace the silica fiber for up to 1200 °C temperature measurement [54].

Up to now, sensors realized based on the above process tend to be large in size, even with sapphire wafer processing, and the device diameter is greater than 1 mm. For small size requirements such as high spatial resolution, a further process development is required.

#### 3.1.4. SiC, Diamond, and Ceramic Diaphragm

Fiber-optic FPs with a diaphragm based on SiC, diamond, and ceramic have also been developed in recent years because of their high melting temperature and Young’s modulus. In 2019, Bae et al. [56] proposed a diamond film-based fiber-optic FP pressure sensor based on dual polymer–ceramic adhesives. In 2016, Jiang et al. [57] realized an all-SiC diaphragm-based fiber-optic pressure sensor using the nickel diffusion bonding technique. In 2022, Liang et al. [58] improved the bonding process and presented an all-SiC fiber-optic FP for applications in a harsh environment. For the ceramic diaphragm, in 2018, Liu et al. [59] proposed a batch-producible fiber-optic FP pressure sensor based on a low-temperature co-fired ceramic technology.

### 3.2. Non-Dielectric Diaphram

#### 3.2.1. Metal Diaphragm

Metals are also commonly used as pressure film materials; thick metal films with large dimensions can be easily processed using computer numerical control (CNC) or polishing technology, and ultra-thin metal films can be realized by the photolithography process. On the other hand, due to the high reflectivity of the metal, it can be easy to achieve good spectral quality in the FP cavity. In addition, the metal has excellent mechanical properties and long-term stability.

**Figure 5:**
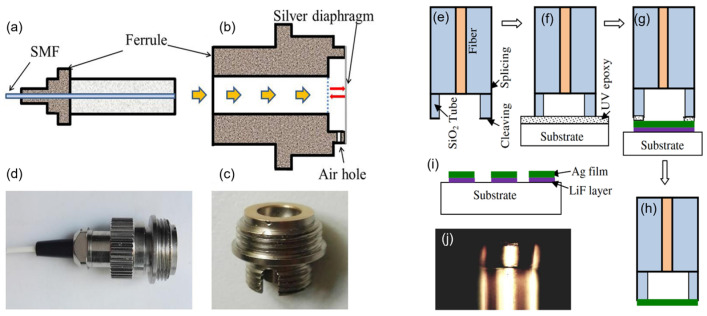
(**a**–**d**) Schematic and photo of thick silver diaphragm-based fiber-optic press sensor. A standard physical contact type of fiber optic connector (**a**) is screwed into the sensor head (**b**,**c**) till the dashed line and then the sensor (**d**) is fabricated. There is an air hole on the sensor head in order to keep the balance of internal and external pressure (reprinted with permission from Ref. [60] © Elsevier, 2017); (**e**–**j**) schematic and photo of thin silver diaphragm (diameter of sensor is 125 μm). Sensor fabrication process (**e**–**h**), silver thin film deposited on a glass substrate (**i**), and an optical microscope picture (**j**) of a typical sensor fabricated (reprinted with permission from Ref. [61] © Optical Society of America).

Typical metal materials used for MEMS fiber-optic FP sensors include gold, silver, and aluminum. The difficulty of processing is due to the assembly of an optical fiber and metal structure. The thermal expansion coefficient mismatch and bonding firmness and airtightness need to be taken into consideration. Traditional processing methods utilize metal to achieve a cavity body and diaphragm and leave an insert hole for optical fiber or ferrule for packaging. The method of fixing is usually adhesive. In 2016, Gong et al. [60] demonstrated a few millimeter lever of a diameter silver diaphragm, as illustrated in Figure 5a–d. The devices prepared by this method are generally mm large in size, but with good structural strength thanks to the metal cavity.

In order to further reduce the size, researchers use optic fiber to realize the cavity and fabricate an ultra-thin metal film based on photolithography. The packaging of the metal diaphragm and cavity body was mainly based on glue [61,62]. Thanks to the photolithography process, a nanometer-scale thick metal film can be realized. Thus, a miniature size, highly sensitive, and quickly responsive fiber-optic FP pressure sensor can be achieved. In 2012, Guo et al. [61] developed a thin silver diaphragm based on the vacuum thermal deposition method, leading to a static pressure sensitivity of 1.6 nm/kPa and a fast pressure response time (resonant frequency ~1.44 MHz), as shown in Figure 5e–j. Xu [62] proposed FP based on a nanothick silver diaphragm by the electroless plating method and demonstrated a pressure sensitivity of 70.5 nm/kPa.

Due to the higher coefficient of thermal expansion of the metal, and, at the same time, the stress caused by the mismatch of the coefficient of thermal expansion between the metal and the silica, as well as the adhesive bonding based on glue, all the above factors adversely affect the application of the metal diaphragm-based sensors.

#### 3.2.2. Polymer Diaphragm

Polymer films are well suited for realizing highly sensitive FP devices, and benefit from their low Young’s modulus and convenient processing methods. Commonly used polymer materials include SU-8 photoresist, PDMS, and UV adhesives, which can be processed by spin-coating transfer, lift-off, and de-molding and curing techniques [63,64,65]. To enhance the mechanical and optical properties of polymer-based diaphragms, researchers have developed composite structures such as metal and polymer multilayers. In 2012, Lai et al. [63] proposed an FP pressure sensor based on a metal and SU-8 photoresist hybrid diaphragm. In 2014, Bae et al. [64] presented a dual-cavity FP sensor composed of a UV-molded cavity covered by a metal/polymer composite diaphragm. In 2015, Eom et al. [65] proposed an optical fiber FP pressure sensor with a lensed fiber and a polymeric diaphragm. Most of the polymer-based FP sensors can easily achieve several hundred nm/kPa to several μm/kPa sensitivity.

However, traditional polymer film processing methods either require multiple complex steps such as spin-coating transfer, or it is difficult to accurately control the film thickness and cavity length. Recently, new fabrication techniques have been induced [66,67,68,69]. In 2017, Zhang et al. [66] presented an FP pressure sensor based on a polyvinyl chloride (PVC) cap created on the end facet of a standard single-mode fiber, accomplishing 65.5 nm/MPa sensitivity. Guggenheim et al. [67] described a novel plano-concave polymer microresonator, constructed by depositing a droplet of optically clear UV-curable liquid polymer onto a dielectric mirror-coated polymer substrate, achieving strong optical confinement, resulting in very high sensitivity. Among the new manufacturing techniques, two-photon polymerization-based 3D printing technology has received the favor of researchers. The fabrication method offers the ability to directly fabricate complex structures on the end face of the optical fiber, with a high degree of freedom and high-precision control, accomplishing high sensitivity and simple processing advantages. Wei et al. [69] proposed a three-dimensional (3D)-printed miniature optical fiber polymer FP pressure sensor in 2020 [24].

However, due to the poor performance of polymer materials in terms of high-temperature resistance and water penetration resistance, they are not very suitable for harsh scenarios such as high temperatures and high pressure and even underwater.

#### 3.2.3. 2D Material Diaphragm

In recent years, two-dimensional (2D) materials have attracted much attention from researchers because of their superior optic, thermal, and mechanical properties. In particular, the atomic-scale ultra-thin thickness of single-layer 2D materials is expected to increase the sensitivity of pressure sensor devices by several orders of magnitude [70,71,72,73,74,75], and thus can be used for the detection of very small and weak pressure.

Common 2D materials include graphene, MoS_2_, etc. Since Ma et al. [70] proposed a graphene membrane-based FP pressure sensor, achieving 39.4 nm/kPa sensitivity, in 2012, graphene has been widely investigated as a sensitive diaphragm in an FP cavity. The bonding of the 2D material to the end face of the FP cavity is usually achieved using wet or dry transfer. In 2015, Li et al. [71,72] systematically investigated the graphene-based fiber-optic FP pressure sensor theoretically. The thickness of a single layer of graphene is only 0.34 nm, and its theoretical sensitivity is as high as 1096 nm/kPa. However, graphene has low reflectivity, with a single layer of graphene having less than 0.1% reflectivity. Graphene–metal composite films can improve reflectivity and enhance FP spectra. In 2019, Dong et al. [73] developed a graphene–silver composite diaphragm-based FP pressure sensor. Meanwhile, the graphene is transferred onto the end face of the FP cavity, and the firmness and bonding airtightness requirements put high demands on the process. Cui et al. [74] induced a (FIB) micromachining and dry exfoliated graphene transfer method and demonstrated a FP sensor from which the air leakage was negligibly small.

**Figure 6:**
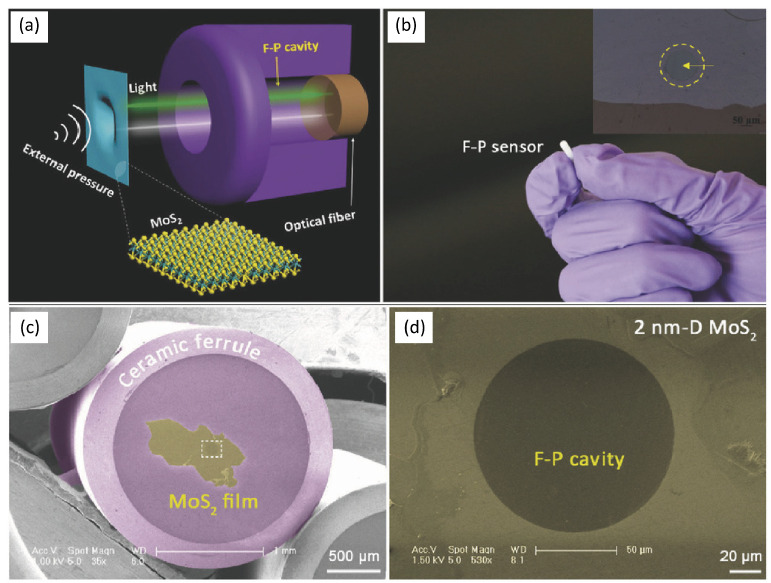
(**a**) Schematic illustration of the FP sensor working mechanism; (**b**) photo of an FP sensor. The inset shows a 2 nm-D MoS_2_ film transferred onto the endface of the FP cavity; (**c**) low-magnification scanning electron microscopy (SEM) image of a ceramic ferrule covered with a piece of 2 nm-D MoS_2_ film; (**d**) the corresponding high-magnification SEM image of the rectangular aera shown in (**c**) (reprinted with permission from Ref. [75] © Wiley, 2016).

Besides graphene, 2D transition metal dichalcogenides (TMDCs) such as MoS_2_-based fiber-optic FP were also investigated. In 2017, Yu et al. [75] proposed ultrasensitive pressure detection of few-layer MoS_2_, with a sensitivity of 89.3 nm Pa^−1^. Figure 6 illustrates the schematic and photograph of the device.

Because of process limitations, it is difficult to realize batch processing of 2D material membrane-based FP pressure sensors. In addition, the 2D material and the FP cavity body are mainly bonded by van der Waals forces, thus their interfacial gas firmness, airtightness, and long-term stability are also a major challenge, which greatly limits their application in high-pressure, liquid, and other environments. Further development of the corresponding bonding technology is needed. Finally, due to the limitation of material properties, it is also difficult to meet the application requirements in high-temperature and strong-vibration environments.

### 3.3. Advantages of Different Diaphragm Materials

Diaphragms based on different materials have their own advantages. Silica diaphragms are compatible with fiber-optic process techniques, providing high temperature and permeation resistance. Silicon, SiC, and diamond-based diaphragms can be processed using the MEMS processes [48,49] and therefore have the ability to be mass-produced. The sapphire diaphragm can work up to extremely high temperatures. On the other side, polymer-based films can easily achieve high sensitivity, while 2D materials can increase the sensitivity by orders of magnitude, maintaining the small size of the device.

## 4. Sensor Applications

### 4.1. Aerodynamic Measurement

Velocity and pressure measurement and distribution analysis of the airflow field have great practical significance in the areas of atmospheric environmental monitoring, aerodynamic studies, turbine maintenance, and navigation control. Fiber-optic airflow sensors have been investigated as alternatives to traditional technologies [14].

For engine monitoring applications, high pressure, high temperatures, and strong vibrations place high demands on the sensor. When the operating temperature is below 1000 °C, all-silicon or silica film-based MEMS FP pressure sensors can be used. In 2004, Zhu et al. [27] presented an all-silica fiber-optic FP pressure sensor by fusion splicing and chemical etching. The 125 µm diameter sensor was applied for dynamic measurements of a turbine engine and showed comparable results with a semiconductor Kulite pressure sensor. When the working temperature is over 1000 °C, only sapphire film-based pressure sensors can survive. In 2013, Pechstedt et al. [76] from Oxsensis Ltd (Didcot, Oxfordshire, UK). demonstrated a fiber-optic pressure sensor based on a multi-cavity design and employed a monolithic sapphire transducer element. The device was designed to measure pressure and temperature simultaneously and was tested over a pressure range of 60 bar and up to 700 °C. The sensor can be used for gas turbine combustor and compressor systems.

**Figure 7:**
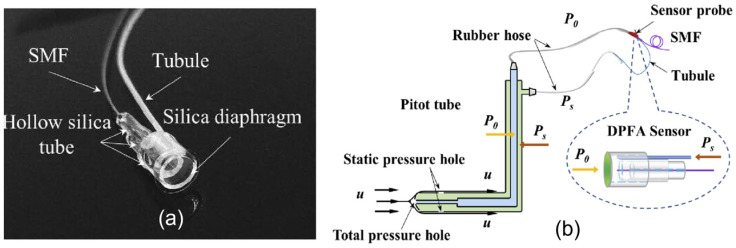
(**a**) Photograph of a DPFA sensor based on FP interferometry. It consists of a standard SMF, fused-silica diaphragm-based FP interferometer, and a plastic tubule; (**b**) schematic diagram of the sensor connected to a Pitot tube for differential pressure measurement. *u* is the airflow velocity, *P_0_* is total pressure in the airflow, *P_s_* is static pressure in the airflow (reprinted with permission from Ref. [77] © Optical Society of America).

For wind tunnel airflow detection, due to the much lower temperature and pressure, silica, silicon, and even metal film-based sensors can be applicable. In 2020, Liu et al. [77] proposed a differential-pressure fiber-optic airflow (DPFA) sensor, with 826.975 nm/kPa sensitivity and 0.008% (0.89 Pa) resolution under a 0~11 kPa measurable range. The sensor was tested in a wind tunnel and successfully measured the airflow velocity of 2.0~119.24 m/s with an accuracy of 0.61%, as presented in Figure 7. Then, in 2022, they developed a three-hole vector probe for high-velocity flow field vector measurement in wind tunnel testing [78]. The device was fabricated based on three fiber-optic tip sensors, offering a new option for turbomachinery research in the future.

### 4.2. Nuclear Power Plants

The faults related to the steam generator tube (SGT) rupture account for 75% of all failure risks in pressurized water reactor nuclear power plants. In 2018, Huang et al. [79] developed a fast fiber FP non-scanning correlation demodulation system (5 kHz), working with FP force and a pressure sensor to assess the Fretting Damage of Steam Generator Tubes.

### 4.3. Underwater Application

**Figure 8:**
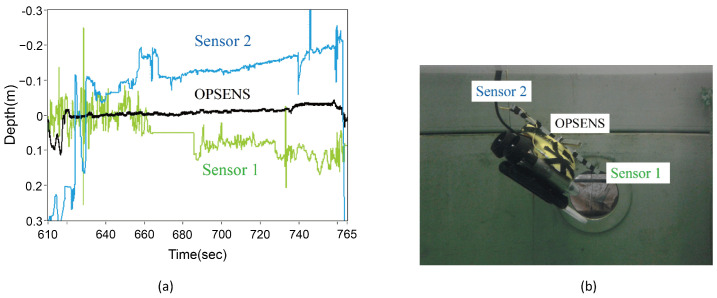
(**a**) Pressure (depth) response of the sensors mounted on the ROV; the sensors 1 and 2 provide different values of the depth (orientation) while data from the OPSENS stays in the middle. The difference in sensors 1 and 2 values is as expected as the ROV was tilted in the horizontal while maintaining a constant depth; (**b**) illustration of sensor installation and ROV orientation (reprinted with permission from Ref. [40] © MDPI, 2017).

Underwater robots are important equipment for marine scientific research. The underwater environment is complex and changeable, so the height and other information are very important for the smooth work and return of the robot. In the face of seawater corrosion and the underwater high-pressure environment, electrical pressure sensors are easily disturbed and damaged. Fiber-optic sensors based on silica can meet the above conditions due to their corrosion resistance and high pressure resistance. In 2012, Lai et al. [63] realized a diaphragm based on metal and SU-8 photoresist for liquid level and specific gravity. In 2017, Duraibabu et al. [40] proposed FP combined with an FBG for remotely operated underwater vehicle (ROV) application, accomplishing 20 nm/kPa pressure sensitivity and 2.5 pm/K temperature sensitivity for compensation. Figure 8 shows the collected data and test setup. In Figure 8a, sensors 1 and 2 show different values of depth, while data from the OPSENS stay in the middle. The data from three sensors are consistent with the attitude of the ROV, as illustrated in Figure 8b.

### 4.4. Medicine and Healthcare

Continuous measurements of pressure within the intracranial, intraocular, and intravascular spaces provide essential diagnostic information for the treatment of traumatic brain injury, glaucoma, and cardiovascular diseases, respectively. In order to reduce the pain of the patient, the instrument channels for vascular intervention or minimally invasive surgery are very narrow; therefore, the size of the sensor is required to be very small. At the same time, the liquid environment of human tissues puts forward high requirements for the safety, corrosion resistance, and anti-interference of the sensor. Fiber-optic-based pressure sensors have not only anti-interference ability, high precision, flexibility, and ease of bending, but are also corrosion-resistant, which well meets the above needs. As it becomes an alternative to electro-mechanical pressure sensors, the optical fiber pressure sensor has become increasingly common in the medical field [28,80,81,82,83,84,85,86,87].

**Figure 9:**
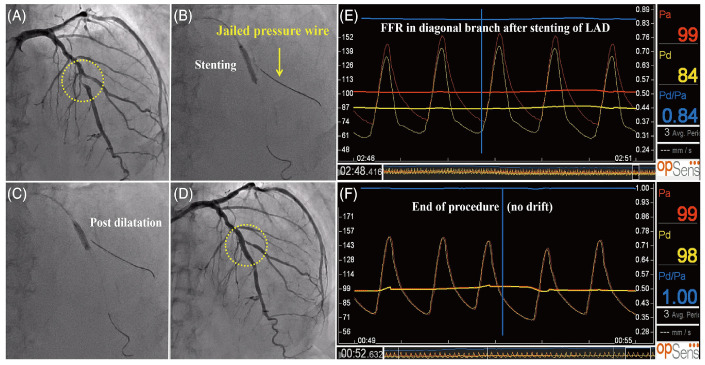
(**A**) Coronary angiography shows a stenotic lesion in the middle of the LAD; (**B**) a stent was deployed with the jailed-pressure wire technique using a durable fiber-optic FP pressure wire; (**C**) post-stenting dilatation was performed using a non-compliant balloon; (**D**,**E**) even though the angiographical stenosis was greater than 50%, additional FKBD was deferred based on the FFR value of 0.84; (**F**) the pressure wire was successfully retracted with slight resistance, and drift phenomenon was not observed. FKBD, final kissing balloon dilatation; FFR, fractional flow reserve; LAD, left anterior descending artery (reprinted with permission from Ref. [86] © Wiley, 2019).

Monitoring of cardiovascular flow rates and pressures during interventional procedures often requires sensors to get inside the blood vessels, and, therefore, requires a sub-mm sized sensor. Sensors based on silica and silicon–glass structures can be fabricated down to 200–125 μm diameter and are suitable for this application. Poeggel et al. [80] demonstrated their cardiovascular pressure application. Wu et al. [81] proposed in vivo blood pressure measurement. In 2014, Poegge et al. [82] demonstrated in vivo Urodynamic Analysis, and Tian et al. [83] proposed an in vivo blood pressure application. Uretsky et al. [85] demonstrated an evaluation of the Strategy of Functionally Optimized Coronary Intervention. Omori et al. [86] investigated FFR measurement, as shown in Figure 9. In 2020, Ulacia et al. [87] finished the First-in-Man O_2_ pilot study.

For cranial pressure monitoring, researchers have tried both minimally invasive implantable and contact methods of use. However, even with non-implantable monitoring, a hole needs to be created in the skull, so the smaller the size of the sensor, the better. In 2013, Cai et al. [28] proposed rat intracranial pressure measurement based on a fiber-optic FP sensor. In 2019, Shin et al. [84] realized monitoring of intracranial pressure and temperature. 

## 5. Discussion

**Figure 10:**
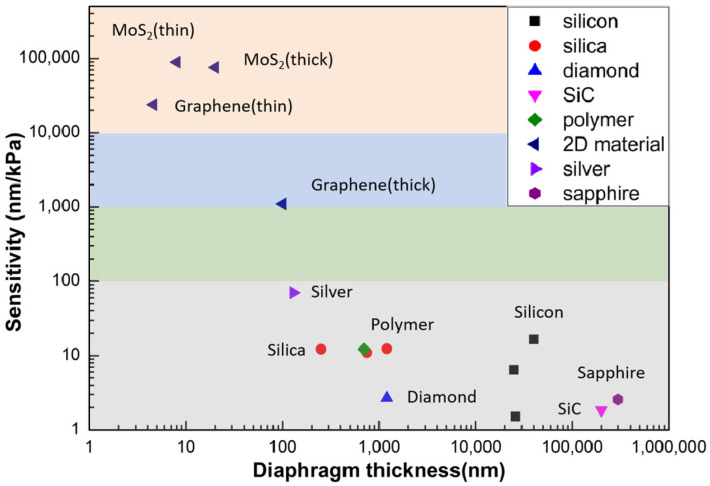
Comparison of the sensitivity of different diaphragm materials (data sources: MoS_2_ films [75], graphene film [70,72], silver film [62], silica film [34,37,38,39], silicon film [44,47,48], polymer film [60], sapphire film [50], SiC film [58], and diamond film [56]).

Table 1 lists the sensitivities of different diaphragm materials, which are also plotted in Figure 10. From the figure, we can see the thinner the diaphragm thickness, the higher the sensitivity. Sensors based on 2D materials have a great advantage in sensitivity, thanks to their nano-scale thickness, which is difficult to achieve with traditional materials and processing methods. Thus, they have great potential for extremely weak pressure detection scenarios. Meanwhile, dielectric materials, such as silica and sapphire, have great value in harsh environment applications, such as those with high temperatures up to thousands of degrees and wide pressure range, etc.

## 6. Conclusions

In summary, MEMS fiber-optic FP pressure sensors demonstrate great application potential in many areas. Researchers have developed a variety of processes, techniques, and different material-based diaphragms to meet the requirements of small size, high accuracy, wide range, and extremely harsh environment measurements.

However, there are still a lot of limitations that need to be solved: (a) most of the silicon-, sapphire-, and SiC-based diaphragms are fabricated by MEMS technology. Limited by process parameters and die-cutting technology, it is difficult to realize ultra-small-size device processing. Meanwhile, silica diaphragms based on fusion processing, which can realize fiber-diameter size sensor components, are difficult to achieve in batch production. In addition, thermal and anodic bonding, as well as the fusion bonding process, will induce high temperatures during processing, which have a great impact on the selection of coating materials, and to a certain extent, also limit the characteristics of the device; (b) at present, most of the work presents bare devices without packaging; (c) most of the work focuses on the response of the device to pressure. Besides temperature, other crosstalk factors that affect device performance have not been adequately investigated.

## 7. Future Directions

In the future, MEMS fiber-optic FP pressure sensors will move more toward harsh and complex environment applications in order to take advantage of their benefits. The main challenges are as follows:

- The fabrication and bonding of diaphragms: further development of processing and bonding technologies is needed to meet the requirements of small-size, high-volume device fabrication for devices of different materials and device specifications.
- Packaging for harsh environments: for future applications, package technology needs to be further developed to meet the harsh scenario requirements and to ensure the long-term stability operation of the devices. When using glue for device encapsulation, the effect of glue and other materials on device performance also needs to be considered.
- Cross-error compensation: it is necessary to further study the mechanism of temperature, strain, bend, and other environmental parameters on the accuracy of the device, and to use appropriate methods to eliminate these errors and improve the overall accuracy of the device.
- The development of more device materials to meet different application requirements.
- Further development of interrogate technology, e.g., improving demodulation speeds, increasing multiplexing capabilities, and finally lowering the cost of the instruments.

## Figures and Tables

**Table 1:** Sensitivities of different diaphragm materials (data sources: MoS_2_ films [75] graphene film [70,72], silver film [62], silica film [34,37,38,39], silicon film [44,47,48], polymer film [60], sapphire film [50], SiC film [58], and diamond film [56]).

Diaphragm Material	Diaphragm Thickness	Pressure Sensitivity
MoS_2_	~8 nm	89,300 nm/kPa
	~20 nm	75,600 nm/kPa
Graphene	~4.6 nm	23,800 nm/kPa
	~100 nm	1100 nm/kPa
Silver film	130 nm	70.5 nm/kPa
Silica film	~250 nm	12.22 nm/kPa
	~750 nm	11 nm/kPa
	1.2 μm	12.4 nm/kPa
	25 μm	0.28 nm/kPa
Silicon film	25 μm	6.408 nm/kPa
	26 μm	1.526 nm/kPa
	40 μm	16.517 nm/kPa
Polymer film	~700 nm	12.2 nm/kPa
Sapphire film	300 μm	2.561 nm/kPa
SiC film	200 μm	1.84 nm/kPa
Diamond film	1.2 μm	2.683 nm/kPa
